# Finerenone: Extending MRAs Prognostic Benefit to the Recently Hospitalized and More Symptomatic Patient with HFpEF

**DOI:** 10.3390/jcm14248730

**Published:** 2025-12-10

**Authors:** Maria Denitza Tinti, Luisa De Gennaro, Raul Limonta, Renata De Maria, Samuela Carigi, Matteo Bianco, Concetta Di Nora, Paolo Manca, Maria Vittoria Matassini, Vittoria Rizzello, Emilia D’Elia, Manuela Benvenuto, Marco Cittar, Geza Halasz, Massimo Iacoviello, Domenico Gabrielli, Furio Colivicchi, Claudio Bilato, Federico Nardi, Massimo Grimaldi, Fabrizio Oliva

**Affiliations:** 1 Cardiology Unit, Azienda Ospedaliera San Camillo—Forlanini, 00152 Rome, Italy; 2 Heart Failure Working Group, ANMCO (Italian National Association of Hospital Cardiologists), 50121 Florence, Italy; luisa.degennaro@policlinico.bari.it (L.D.G.); renata_de_maria@hotmail.com (R.D.M.); samuela.carigi@auslromagna.it (S.C.); m.bianco@sanluigi.piemonte.it (M.B.); concetta.dinora@asufc.sanita.fvg.it (C.D.N.); pmanca@ismett.edu (P.M.); mariavittoria.matassini@ospedaliriuniti.marche.it (M.V.M.); vrizzello@hsangiovanni.roma.it (V.R.); 3 Cardiology Department, Azienda Ospedaliera Universitaria Consorziale Policlinico di Bari, 70124 Bari, Italy; 4 School of Cardiovascular Disease, Milano-Bicocca University, 20126 Milan, Italy; 5 Department of Primary Care, ASST Fatebenefratelli Sacco, 20131 Milan, Italy; 6 Cardiology Unit, Infermi Hospital, 47923 Rimini, Italy; 7 Division of Cardiology, Azienda Ospedaliera Universitaria San Luigi Gonzaga, 10043 Orbassano, Italy; 8 Department of Cardiothoracic Science, Azienda Sanitaria Universitaria Integrata di Udine, 33100 Udine, Italy; 9 Department of Clinical Cardiology and Heart Failure, Mediterranean Institute for Transplantation and Advanced Specialized Therapies, ISMETT IRCCS, 90121 Palermo, Italy; 10 Cardiac Intensive Care Unit-Cardiology Division, Department of Cardiovascular Sciences, Azienda Ospedaliera Universitaria delle Marche, 60124 Ancona, Italy; 11 Cardiology Unit, Azienda Ospedaliera San Giovanni—Addolorata, 00184 Rome, Italy; 12 Cardiovascular Department, Azienda Ospedaliera Papa Giovanni XXIII, 24127 Bergamo, Italy; 13 Cardiology Unit, Ospedale Civile G. Mazzini, 64100 Teramo, Italy; 14 Cardiovascular Pathology Unit, Territorial Specialist Department, Azienda Sanitaria Universitaria Giuliano-Isontina (ASUGI), 34148 Trieste, Italy; 15 University Cardiology Unit—UTIC, Policlinico Riuniti, 71100 Foggia, Italy; 16 Heart Care Foundation, 50121 Florence, Italy; m.grimaldi@miulli.it; 17 Clinical and Rehabilitation Cardiology Division, San Filippo Neri Hospital—ASL Roma 1, 00135 Roma, Italy; 18 Cardiology Unit, Ospedali dell’Ovest Vicentino, Azienda ULSS 8 Berica, 36100 Vicenza, Italy; 19 Cardiology Department, Santo Spirito Hospital, 15033 Casale Monferrato, Italy; 20 U.O.C. Cardiologia-UTIC, Ospedale Miulli, 70021 Acquaviva delle Fonti, Italy; 21 ANMCO Presidency (Italian National Association of Hospital Cardiologists), 50121 Florence, Italy; 22 Intensive Cardiac Care Unit, De Gasperis Cardio Center, ASST Grande Ospedale Metropolitano Niguarda, 20162 Milan, Italy

**Keywords:** HFpEF, HFmrEF, finerenone, mineralcorticoid receptor antagonists

## Abstract

Mineralocorticoid receptor antagonism represents a therapeutic cornerstone in patients with HFrEF. However, the efficacy of MRAs in patients HFmrEF or HFpEF remains unclear. While the TOPCAT trial did not demonstrate a statistically significant reduction in cardiovascular mortality or hospitalizations for HF in this patient population, post hoc data suggested potential clinical benefits of MRAs in specific patient subgroups. More recently, the landscape has evolved with the FINEARTS-HF trial, which generated novel evidence regarding finerenone, a non-steroidal MRA, in the HFmrEF and HFpEF cohorts. This investigation established a statistically significant decrease in the primary composite endpoint, comprising cardiovascular death and worsening heart failure events. This positive outcome was principally attributable to a lower incidence of total WHF events. Key findings also highlighted the therapy’s rapid onset of action and favorable safety profile. Notably, finerenone’s benefit was consistent across the entire LVEF spectrum and was not influenced by baseline SGLT2i use, sex, or age. Finerenone’s established indication is for patients with type 2 diabetes mellitus and chronic kidney disease to reduce heart failure risk. However, based on the findings of the FINEARTS-HF trial, finerenone may represent a novel therapeutic pillar for patients with HFpEF and HFmrEF. In this review, we aim to describe the potential benefits of MRAs in the pathophysiology of HFpEF and HFmrEF and in different patient phenotypes, the results of the FINEARTS-HF trial, and the most important subanalyses of the study.

## 1. Introduction

Until recently, patients with heart failure with preserved ejection fraction (HFpEF) lacked disease-modifying treatments. For many years, management centered on alleviating symptoms, primarily with diuretics, and addressing the patient’s coexisting comorbidities. Only recently sodium-glucose co-transporter 2 inhibitors (SGLT2i), originally developed as glucose-lowering agents, gained approval for reducing mortality and heart failure (HF) hospitalizations across the entire spectrum of left ventricular ejection fraction (LVEF) [1,2]. A central pathophysiological mechanism in heart failure is the upregulation of mineralocorticoid receptor (MR) signaling, driven by dysregulation of the renin–angiotensin–aldosterone system (RAAS). Mineralocorticoid receptor antagonists (MRAs) are a cornerstone of evidence-based therapy for heart failure with reduced ejection fraction (HFrEF) [1,3,4], with a significant effect on adverse cardiac remodeling [5]. For patients with HFpEF or mildly reduced ejection fraction (HFmrEF), however, the therapeutic benefit of this pathway is less well established. Based on this background the TOPCAT trial (Treatment of Preserved Cardiac Function Heart Failure with an Aldosterone Antagonist) evaluated the efficacy of spironolactone, the first steroidal MRA used in HF, in patients with a left ventricular ejection fraction (LVEF) ≥ 45% [6]. The study did not achieve statistical significance for its primary composite endpoint. However, the enrollment criteria and the heterogeneity of the study population likely influenced the trial outcomes [7].

For this reason, there is growing interest in a new class of MRAs, which, unlike the first-generation agents, are nonsteroidal in structure. Finerenone, the first drug in this class, has already been shown to slow the progression of chronic kidney disease (CKD) and improve cardiovascular outcomes in patients with CKD and diabetes [8,9]. Recently, the results of the FINEARTS-HF trial (Finerenone in patients with heart failure with mildly reduced or preserved ejection fraction) were published, evaluating its efficacy in patients with HFmrEF or HFpEF [10,11].

In this review, we aim to provide an overview of the pathophysiological rationale for mineralocorticoid receptor antagonism in HFpEF and HFmrEF, integrating evidence from the recent FINEARTS-HF trial within the broader context of HF management. We discuss the efficacy of finerenone across different patient phenotypes and comorbidities, analyzing the transition from steroidal to non-steroidal MRAs and clinical insights to guide patient selection and implementation in daily practice.

## 2. The Pathogenic Role of the Mineralocorticoid Receptor and the Rational for Antagonism

Overactivation of the MR by aldosterone starts deleterious cellular responses, including endothelial dysfunction, myocardial hypertrophy, apoptosis, and fibrosis. The MR is also a cardiac inflammation mediator; its activation can induce the transformation of macrophages into myofibroblasts, a key point in the myocardial fibrotic and inflammatory cascade. This sustained inflammatory environment directly promotes tissue injury and perpetuates a self-amplifying cycle of fibrotic remodeling [12].

**Figure 1:**
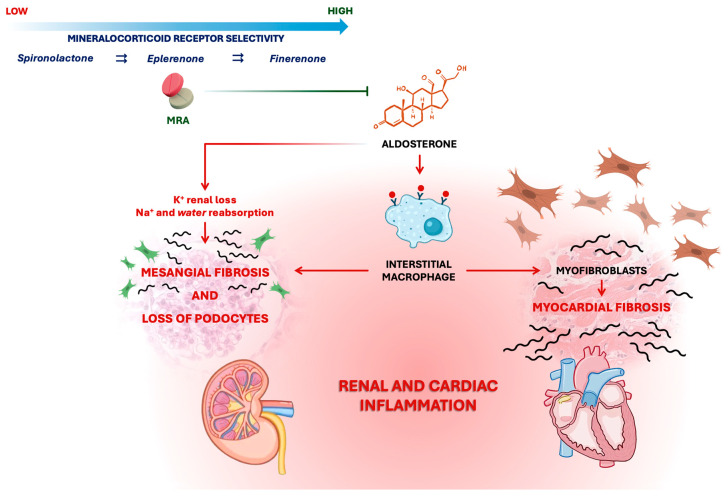
Pathophysiologic role of inflammation and fibrosis in heart failure with preserved ejection fraction.

In the kidney, MR activation promotes sodium retention and potassium excretion, which can lead to mesangial fibrosis and podocyte loss, culminating in progressive kidney dysfunction [13]. Thus, MR activation plays a central role in cardiorenal disease, translating diverse pathological insults, such as hypertension and metabolic stress, into a common downstream pathway of inflammation and fibrosis. This makes the MR a target for therapeutic intervention (Figure 1).

Since the publication of the RALES trial, it has become evident that a therapeutic target of MRAs in HF is fibrosis. A key subgroup analysis from that study revealed that the clinical benefit of spironolactone was most significant in patients exhibiting markers of active collagen turnover, which were substantially reduced by the therapy [14]. The anti-fibrotic effects of MRAs have since been consistently demonstrated across the whole spectrum of cardiovascular conditions, where they inhibit the synthesis of both type I and type III collagen [15,16,17]. Thus, MRAs offer a direct mechanism to counteract cardiovascular fibrosis, adverse remodeling, and inflammation.

Pharmacological agents targeting the MR can be classified into two main categories, steroidal and non-steroidal, distinguished by their chemical structures, pharmacological characteristics, and clinical profiles.

### 2.1. Steroidal MRAs

The first- and second-generation agents, spironolactone and eplerenone, constitute the class of steroidal MRAs (sMRAs). Their molecular framework is built upon a core steroidal backbone, which is structurally similar to endogenous hormones. This allows for effective MR binding and antagonism, but with poor receptor selectivity. Spironolactone, in particular, interacts with androgen and progesterone receptors, leading to a hormonal adverse effects such as gynecomastia, menstrual irregularities, and impotence. Eplerenone, a more selective second-generation sMRA, was developed to mitigate these issues. While it has a significantly lower affinity for other steroid receptors, this enhanced selectivity is accompanied by a reduced binding affinity for the MR itself [18].

The pharmacokinetic properties of these two agents are different. Spironolactone has a short plasma half-life of about 1.4 h but is converted in the liver to several active metabolites, such as canrenone, which have much longer half-lives (e.g., ~16.5 h). These metabolites are responsible for the drug’s sustained action but also prolong the risk of adverse events like hyperkalemia [19]. Eplerenone, by contrast, has a half-life of 3 to 6 h and lacks active metabolites, resulting in a more predictable pharmacological profile [20].

A key pharmacological characteristic of sMRAs is their preferential concentration in the kidneys compared to the heart. This renal concentration may amplify their diuretic effects but also the risk of hyperkalemia by potently inhibiting MR-mediated potassium excretion [21]. The combination of hormonal side effects and hyperkalemia risk, especially in patients with CKD, has led to high rates of treatment discontinuation and underutilization in clinical practice [22,23].

### 2.2. Non-Steroidal MRAs

The development of non-steroidal MRAs (nsMRAs), including agents like finerenone, apararenone, and esaxerenone, is a step forward in targeting the MR axis. These drugs have a chemical structure fundamentally different from endogenous steroids, allowing for high-potency and high-affinity binding to the MR without interacting with other steroid hormone receptors. This exceptional selectivity effectively eliminates the risk of hormonal side effects. Finerenone is the most extensively studied agent in this class [18].

NsMRAs also offer a more favorable pharmacokinetic profile, characterized by a short duration of action (finerenone’s half-life is 2–3 h) and an absence of active metabolites, leading to a more predictable pharmacodynamic effect. Furthermore, unlike sMRAs, finerenone demonstrates a balanced distribution between cardiac and renal tissues. This allows for potent anti-inflammatory and anti-fibrotic effects in the heart while avoiding excessive accumulation in the kidneys, which is hypothesized to mitigate the risk of hyperkalemia [24].

### 2.3. Mineralocorticoid Receptor Axis Modulation

Two innovative therapeutic strategies are currently in development: mineralocorticoid receptor modulators (MRMs) and aldosterone synthase inhibitors (ASIs) [25].

MRMs are designed to interact with the MR in a tissue-selective manner. The goal is to achieve antagonism in tissues where MR activation is pathological (e.g., heart, vessels, the kidney) while having a minimal effect on tissues where MR blockade can be counterproductive, such as the renal epithelial cells responsible for potassium excretion. This approach aims to uncouple the desired protective effects from adverse impacts on electrolyte balance. Balcinrenone is a leading MRM in clinical development, and trials like MIRO-CKD [26] and MIRACLE [27] are assessing its potential to provide a superior benefit-risk ratio in patients with CKD and HF.

Aldosterone synthase inhibitors (ASIs) employ an upstream blockade strategy by directly inhibiting the enzyme responsible for aldosterone production. This approach is meant to reduce both circulating and local aldosterone levels, thereby preventing MR activation across all tissues. By preserving basal MR activity mediated by glucocorticoids, ASIs may offer organ protection with a reduced risk of hyperkalemia. Early clinical trials with novel ASIs like baxdrostat and lorundrostat have shown promising reductions in blood pressure and aldosterone concentrations in patients with resistant hypertension, laying the groundwork for large-scale Phase III trials [28].

## 3. From Subgroup Analyses to a Phenotypic Definition of MRA-Responsive Patients: Lessons Learned from TOPCAT

The TOPCAT trial (Treatment of Preserved Cardiac Function Heart Failure with an Aldosterone Antagonist), published in 2014, was the first major study designed to evaluate spironolactone’s efficacy in patients with HFpEF and HFmrEF using a mineralocorticoid receptor antagonist approach [6].

TOPCAT was designed to assess whether spironolactone could reduce a composite primary endpoint of cardiovascular death, resuscitated cardiac arrest, or HF hospitalization in patients with HFpEF (LVEF ≥ 45%) and either a history of HF hospitalization or elevated natriuretic peptides. The study enrolled 3445 patients across the US, Canada, Argentina, Brazil, Russia, and Georgia. It yielded a neutral result for the primary composite endpoint (Hazard Ratio [HR] 0.89, 95% CI 0.77–1.04; *p* = 0.14).

However, doubts emerged about the reliability of these results. Notably, the number of primary events was unexpectedly higher in patients enrolled based on natriuretic peptides than in those enrolled due to prior HF hospitalization, a surprising outcome given the latter group’s presumed higher risk [29,30]. Most patients with recent HF hospitalizations were enrolled in Russia and Georgia, suggesting these patients had a lower-risk phenotype more similar to hypertensive heart disease. Conversely, among patients enrolled in the Americas, event rates in the placebo group aligned more closely with prior HFpEF studies, and spironolactone significantly reduced the primary endpoint (HR 0.82, 95% CI 0.69–0.98; *p* = 0.026) and its individual components. A subsequent analysis confirmed these regional inconsistencies [31].

Given these issues and post hoc findings, ACC/AHA guidelines currently give a class IIb recommendation for MRAs in appropriately selected symptomatic HFpEF patients (elevated BNP or recent HF hospitalization, eGFR > 30 mL/min/1.73 m^2^, serum creatinine < 2.5 mg/dL, and serum potassium < 5.0 mEq/L), especially those at the lower end of the LVEF spectrum [32]. In contrast, European guidelines recommend MRAs only for patients with HFmrEF, not HFpEF [1].

TOPCAT’s inconclusive primary findings prompted several post hoc analyses aimed at identifying specific HFpEF or HFmrEF patient subtypes more likely to benefit from MRA therapy.

Spironolactone’s effects showed significant interaction between treatment arm and primary endpoint across the LVEF spectrum; patients with lower LVEF derived more benefit, supporting the hypothesis that spironolactone’s efficacy in HFpEF may be linked not only to antifibrotic properties but also to modulation of neurohormonal activation, particularly relevant in cases of borderline reduced LVEF [33]. No interaction was found between sex and treatment for the primary composite endpoint, but spironolactone was associated with reduced all-cause mortality in women, with a significant interaction by sex [34]. The treatment effect remained stable across the LVEF spectrum in women but varied in men, who showed greater benefit only at lower LVEF levels, akin to HFrEF patients [35]. This may relate to sex-based differences in ventricular geometry and function: women tend to have smaller ventricular dimensions and higher LVEF, so an LVEF of 50–55% in women might already represent subclinical systolic dysfunction.

Spironolactone consistently reduced the primary endpoint across renal function strata, but the risk of drug discontinuation increased with decreasing eGFR, indicating reduced tolerability in advanced CKD: event rates and adverse events were nearly twice as high in those with estimated glomerular filtration rate (eGFR) < 45 mL/min/1.73 m^2^ compared to those with preserved renal function [36]. Also, worsening renal function (WRF), more frequent with spironolactone than placebo, was associated with a higher incidence of the primary endpoint [37]. However, among patients who experienced WRF, those receiving spironolactone had lower cardiovascular and all-cause mortality rates than those on placebo.

These findings underscore the importance of monitoring renal function and potassium levels, especially in older or CKD patients receiving MRAs. While no significant interaction was found between age and efficacy for the composite endpoint, older patients were more likely to discontinue spironolactone, mainly due to hyperkalemia or worsening renal function [38].

More systematic approaches to define phenotypes that may benefit most from MRA therapy have been prompted: a cluster analysis of eight clinical variables (age, sex, race, diabetes, atrial fibrillation, obesity, New York Heart Association (NYHA) class III/IV symptoms, and CKD) in the entire TOPCAT cohort identified three distinct phenogroups: (1) Younger patients with mild symptoms; (2) Older patients with reduced ventricular volumes and atrial fibrillation; (3) Obese patients with diabetes and advanced symptoms [39]. Spironolactone was more effective in phenogroup 3, significantly reducing the risk of the primary endpoint (*p* for interaction = 0.016) and HF hospitalization (*p* for interaction = 0.007). Clues to the greatest benefit observed in this phenogroup include elevated markers of metabolic dysregulation and systemic inflammation, heightened RAAS activity, non-alcoholic fatty liver disease/fibrosis, and CKD.

These results reinforce the notion that HFpEF is a heterogeneous syndrome; obese patients with impaired natriuresis due to RAAS hyperactivation appear to benefit the most from MRA therapy.

While TOPCAT provided important signals, the clinical application of spironolactone in this population has been hindered by its side effect profile—specifically hormonal adverse events and the risk of hyperkalemia—and the lack of definitive efficacy data. This unmet need paved the way for the development of non-steroidal MRAs (nsMRAs). Unlike their steroidal counterparts, nsMRAs such as finerenone are characterized by high potency, distinct receptor selectivity, and a balanced tissue distribution between the heart and kidneys. These properties formed the basis for the FINEARTS-HF trial, aiming to extend the prognostic benefits seen in diabetic kidney disease to the broader HFmrEF and HFpEF population.

## 4. Finerenone and the FINEARTS-HF Trial: From Evidence of Efficacy in Diabetic Nephropathy to Its Role in HFpEF and HFmrEF

The distinct pharmacokinetic and pharmacodynamic characteristics of finerenone, combined with its favorable safety profile, provided the rationale for the design of two parallel clinical trials, conducted in patients with CKD and diabetes mellitus: FIDELIO-DKD (Finerenone in Reducing Kidney Failure and Disease Progression in Diabetic Kidney Disease) and FIGARO-DKD (Finerenone in Reducing Cardiovascular Mortality and Morbidity in Diabetic Kidney Disease). The trials targeted different endpoints within the shared population of patients with diabetic kidney disease. The former study was designed to assess renal outcomes; the latter, by contrast, focused on cardiovascular events [8,9].

Briefly, the FIDELIO-DKD trial enrolled over 5000 patients with type 2 diabetes and CKD with a history of diabetic retinopathy or more severe albuminuria and eGFR between 25 and 75 mL/min/1.73 m^2^. Finerenone significantly reduced the primary composite endpoint (≥40% decline in eGFR from baseline or renal death) by 18% compared with placebo (HR 0.82; 95% CI, 0.73–0.93; *p* = 0.001), and the key secondary cardiovascular composite endpoint (cardiovascular death, nonfatal myocardial infarction, nonfatal stroke, or hospitalization for heart failure) by 14% (HR 0.86; 95% CI, 0.75–0.99; *p* = 0.03), though with a higher incidence of hyperkalemia (2.3% vs. 0.9%) [8].

In FIGARO-DKD, over 7000 patients with earlier-stage CKD than those in FIDELIO-DKD were randomized to receive oral finerenone or placebo. Patients with HFrEF were excluded since MRAs are already indicated in this setting. The primary endpoint, a composite of cardiovascular death, nonfatal myocardial infarction, nonfatal stroke, or hospitalization for HF, occurred in 12.4% of the finerenone group and 14.2% of the placebo group (HR 0.87; 95% CI, 0.76–0.98; *p* = 0.03), driven largely by a 29% reduction in HF hospitalizations (HR 0.71; 95% CI, 0.56–0.90). The secondary composite renal endpoint occurred in 9.5% of the finerenone group and 10.8% of the placebo group (HR 0.87; 95% CI, 0.76–1.01; *p* = 0.07). Finerenone thus improved cardiovascular outcomes in patients with mild-to-moderate CKD and type 2 diabetes receiving optimized RAAS blockade, across all eGFR and urinary albumin-to-creatinine ratio (UACR) categories [9].

To assess comprehensive cardiovascular and renal outcomes, the “FIDELITY” pooled analysis was conducted by integrating data from both FIDELIO-DKD and FIGARO-DKD. The cardiovascular endpoint comprised CV death, nonfatal MI, nonfatal stroke, or HF hospitalization. The composite renal outcome included kidney failure, persistent eGFR < 15 mL/min/1.73 m^2^, ≥57% sustained decline in eGFR, renal death, dialysis initiation, or kidney transplantation [40]. The analysis, which included 13,026 patients followed for a median of 3 years, confirmed finerenone’s efficacy. The composite cardiovascular endpoint was reduced by 14% (HR 0.86; 95% CI, 0.78–0.95; *p* = 0.0018). A significant reduction of 23% was also observed for the composite renal endpoint (HR 0.77; 95% CI, 0.67–0.88; *p* = 0.0002). This benefit was accompanied by a higher incidence of hyperkalemia requiring treatment cessation (1.7% vs. 0.6%).

Based on this rationale, the FINEARTS-HF trial (Finerenone Trial to Investigate Efficacy and Safety Superior to Placebo in Patients with Heart Failure) was designed to assess finerenone’s efficacy in the HFmrEF and HFpEF populations [10]. The study enrolled patients with LVEF ≥ 40%, in NYHA class II–IV, with elevated natriuretic peptides (NT-proBNP ≥ 300 pg/mL or BNP ≥ 100 pg/mL), serum potassium < 5.0 mmol/L and eGFR > 25 mL/min/1.73 m^2^. Key exclusion criteria included systolic BP > 160 mmHg or a recent history (preceding 90 days) of myocardial infarction or other events that could reduce LVEF. Randomization to placebo or finerenone involved dosing stratified by baseline eGFR (max 20 mg/day for eGFR ≤ 60; max 40 mg/day for eGFR ≥ 60 mL/min/1.73 m^2^). To capture the clinically relevant burden of disease, the primary endpoint was a composite of cardiovascular death or total HF-related events (first and recurrent hospitalizations or urgent visits) over a 42-month follow-up. Beyond the primary composite endpoint, the trial was designed to evaluate a broad spectrum of secondary efficacy and safety signals. These included the cumulative burden of heart failure events and death from any cause. Functional status was assessed via changes in NYHA class, with a specific focus on improvement from baseline to week 52. Patient-reported health status was tracked using the Kansas City Cardiomyopathy Questionnaire (KCCQ) Total Symptom Score at 6, 9, and 12 months. Furthermore, a composite renal endpoint was adjudicated to monitor safety, defined by a sustained decline in eGFR of ≥50%, a drop in eGFR below 15 mL/min/1.73 m^2^, or the initiation of dialysis or renal transplantation.

Over a 28-month period, the trial enrolled a global cohort of 6014 patients from 37 countries. This population had a mean age of 72 ± 10 years, 45% were women, and the majority (69%) presented with NYHA class II symptoms. Echocardiographic data revealed a mean baseline LVEF of 53 ± 8%; notably, however, the inclusion criteria permitted a specific subgroup of 271 patients (4.5%) with a documented history of LVEF < 40% (HFimpEF) [10].

In contrast to FIDELIO-DKD and FIGARO-DKD, FINEARTS-HF also included non-diabetic patients with a broad range of kidney function [41]. A significant portion of the cohort (48%) had an eGFR < 60 mL/min/1.73 m^2^ at enrollment, and the median eGFR for the study group was 61 (IQR 47–77) mL/min/1.73 m^2^. Median UACR was 18 (IQR 7–67) mg/g; 39% had UACR > 30 mg/g. Based on KDIGO criteria, 35% of patients were at low risk, 29% at moderate, 20% at high, and 16% at very high risk. UACR is rarely assessed in HF trials, but adding UACR to eGFR enabled reclassification into higher KDIGO risk categories for 2167 (37%) participants [42].

Eligibility criteria were similar to recent HFpEF/mrEF trials [43,44], but the FINEARTS-HF population was at relatively higher risk: Over half of the patients (54%) had an HF-related event within the three months preceding inclusion, and a significant subgroup (20%) was enrolled within one week of an HF hospitalization. Most patients received guideline-directed medical therapy for HF, including beta-blockers (85%), angiotensin–converting enzyme (ACE) inhibitors (36%), angiotensin II receptor blockers (ARBs) (44%), angiotensin receptor–neprilysin inhibitors (ARNIs) (9%), calcium channel blockers (33%), and SGLT2 inhibitors (14%). When compared to other contemporary studies in this population, the FINEARTS-HF trial reported the highest implementation rates for SGLT2is and ARNIs, even though the absolute percentages were modest.

Finerenone significantly reduced the primary composite endpoint of cardiovascular death and total worsening heart failure (WHF) events by 16% (Rate Ratio [RR] 0.84; 95% Confidence Interval [CI], 0.74–0.95; *p* = 0.007). This benefit was primarily driven by a robust reduction in total WHF events (RR 0.82; 95% CI, 0.71–0.94; *p* = 0.006). When analyzing the components using a time-to-first-event model, finerenone was associated with a 16% relative risk reduction in the composite outcome.

Regarding secondary outcomes, cardiovascular death occurred in 8.1% of patients in the finerenone group versus 8.7% in the placebo group (HR 0.93; 95% CI, 0.78–1.11), a difference that was not statistically significant. However, finerenone demonstrated a significant improvement in patient-reported health status: the KCCQ-TSS score improved by a mean of 1.6 points more in the treatment arm compared to placebo (*p* < 0.001).

The safety analysis confirmed the distinct profile of this non-steroidal agent. Hyperkalemia events occurred more frequently with finerenone (9.7% vs. 2.4%), but severe hyperkalemia (>6.0 mmol/L) was uncommon (3.0% vs. 1.4%), and importantly, there were no hyperkalemia-related deaths.

Regarding survival, death from any cause occurred in 16.4% of patients in the treatment arm compared to 17.4% in the control group. This difference did not achieve statistical significance. Likewise, the analysis of the primary renal composite outcome—defined as a sustained decline in eGFR of greater than 50%, progression to end-stage kidney disease, or the need for dialysis or transplantation—revealed no significant disparity between the two groups.

The authors attributed this neutral renal finding to the population’s low baseline risk of renal progression and low albuminuria prevalence. In terms of specific renal markers, finerenone therapy was associated with a marked protective effect against albuminuria, reducing the risk of new-onset microalbuminuria by 24% (HR 0.76; 95% CI 0.68–0.83) and macroalbuminuria by 38% (HR 0.62; 95% CI 0.53–0.73). This benefit occurred despite a documented initial, reversible dip in eGFR of −2.9 mL/min/1.73 m^2^ (95% CI −3.4 to −2.4) observed in the treatment arm [45].

Safety assessments revealed that elevations in both serum creatinine and potassium were more prevalent among patients assigned to finerenone. Severe hyperkalemia, defined by potassium levels > 6.0 mmol/L, were recorded in 3.0% of the active treatment arm compared with 1.4% in the control group. Notably, however, no fatalities related to hyperkalemia were observed throughout the study.

## 5. Clinical Phenotypes and Treatment Response: Insights from FINEARTS-HF Subanalyses

The FINEARTS-HF trial included numerous prespecified analyses that provide critical guidance for clinical practice. Estimates of lifetime benefit suggest that the intervention might provide up to three additional years of event-free survival, a finding that underscores the long-term value of early initiation [46]. Unlike prior HFpEF trials, which often showed heterogeneous responses, finerenone demonstrated a remarkably consistent benefit across a broad spectrum of patient characteristics.

### 5.1. Impact of Left Ventricular Ejection Fraction

Whereas prior trials indicated diminished efficacy of neurohormonal blockade at higher LVEF values, finerenone demonstrated consistent benefits across the LVEF spectrum, reducing the risk of cardiovascular death and worsening HF events in any given LVEF category (LVEF < 50%: RR 0.84, 95% CI 0.68–1.03; LVEF 50–59%, RR 0.80, 95% CI 0.66–0.97; LVEF ≥ 60%, RR 0.94, 95% CI 0.70–1.25; *p* for interaction = 0.70) [47].

This divergence from the typical response seen with other neurohormonal antagonists might be driven by unique cohort characteristics. Specifically, 42% of the cohort was enrolled soon after an acute decompensation, and the substantial neurohumoral activation—evidenced by elevated natriuretic peptides even in patients with preserved LVEF—likely rendered this population particularly responsive to MR antagonism.

However, the lack of any interaction between the effect of finerenone and baseline NT-proBNP levels argues against this hypothesis. Finerenone led to an early and sustained reduction in NT-proBNP concentrations and baseline NT-proBNP levels did not modify the effect of finerenone on the primary outcome (*p* for interaction = 0.92) [48]. This lack of interaction contrasts with findings from the TOPCAT trial, where diminished benefit was observed in patients with higher natriuretic peptide levels [49].

Even in the specific subgroup of patients with heart failure with improved ejection fraction (HFimpEF), the efficacy was consistent; approximately 5% of patients enrolled in FINEARTS-HF had a history of LVEF < 40%, a lower proportion than that reported in the DELIVER trial [50]. The effect of finerenone on the primary endpoint was similar between patients with HFimpEF and those with consistently preserved LVEF (*p* for interaction = 0.36) [51]. Due to their higher baseline risk, patients with HFimpEF experienced greater absolute risk reduction (9.2 vs. 2.5 events per 100 person-years). While patients with HFimpEF appeared more prone to hypotension during treatment, the overall safety profile of finerenone was comparable to that of patients without prior reduced LVEF. These findings support the integration of finerenone into the therapeutic strategy for HFimpEF, alongside other disease-modifying agents such as SGLT2is. Even when LVEF values return to a normal range, individuals with HFimpEF are not healed; they keep a heightened susceptibility to clinical events driven by enduring myocardial structural and functional impairments, reinforcing the need for ongoing guideline-directed medical therapy [52].

### 5.2. Impact of Patient Characteristics and Frailty

A key finding is the stability of finerenone’s efficacy regardless of age or sex. While women in the trial were older and more symptomatic than men, the relative risk reduction was comparable between sexes (HR 0.78 for women vs. 0.88 for men; *p*-interaction = 0.41); Moreover, the safety profile was comparable [53].

Similarly, efficacy was maintained across all age quartiles, suggesting that advanced age should not be a barrier to MRA initiation [54]. Even frailty, a common condition in HFpEF that often complicates therapy, did not modify the safety or efficacy of the drug (*p*-interaction = 0.77) [55].

Finerenone demonstrated consistent benefit across baseline KCCQ-TSS [56] and NYHA functional classes, with no statistically significant interaction by NYHA class (*p* for interaction = 0.54). However, absolute risk reductions were greater in patients with NYHA class III/IV (absolute rate reduction [ARR] of 4.5 per 100 person-years) compared to class II (ARR of 2.0 per 100 person-years) [57]. These findings contrast with prior studies in HFrEF, which have shown that therapeutic efficacy may be attenuated in patients with advanced functional class, likely due to the greater burden of cardiovascular and non-cardiovascular comorbidities. Such multimorbidity is even more prevalent in individuals with HFmrEF and HFpEF, potentially amplifying this issue.

Notably, obesity emerged as the only significant effect modifier. Patients with a higher body mass index (BMI) derived greater benefit from finerenone (*p*-interaction = 0.005 when analyzed as a continuous variable). This finding aligns with the pathophysiological understanding that visceral adiposity drives aldosterone production independent of the RAAS cascade, potentially making obese phenotypes particularly responsive to MR antagonism [58].

### 5.3. Efficacy Across Comorbidities

The trial confirmed that finerenone’s benefit is robust even in the presence of major non-cardiovascular comorbidities.

**Renal Function:** Benefits were consistent across all baseline kidney risk categories for the primary endpoint (*p*-interaction = 0.24). While the drug did not significantly alter the eGFR slope in this lower-risk population compared to diabetic kidney disease trials [59], it significantly reduced new-onset albuminuria [45]; the safety profile of finerenone remained favorable across the risk spectrum. Adverse safety events, including hyperkalemia, were not amplified among participants with higher baseline kidney risk when compared to placebo [60].

**Atrial Fibrillation (AF):** Atrial fibrillation has previously been associated with attenuated therapeutic responses in HFrEF to agents such as β-blockers and cardiac resynchronization therapy. To date, SGLT2i inhibitors are the only class to have demonstrated unequivocal benefit in patients with concomitant AF and heart failure. In FINEARTS-HF, the effect of finerenone on the primary outcome was consistent across patients with and without AF, as well as across AF subtypes (RR 0.80; 95% CI, 0.65–0.98 with no AF; RR 0.83; 95% CI, 0.65–1.06 with paroxysmal AF; and RR 0.85; 95% CI, 0.69–1.05 with persistent/permanent AF; *p* for interaction = 0.94) [61]. Additionally, a numerical reduction in new-onset AF or atrial flutter was observed with finerenone compared to placebo (HR 0.77; 95% CI, 0.57–1.04; *p* = 0.09). Given the potential contribution of aldosterone to atrial remodeling and AF development, this trend, despite falling short of statistical significance, may suggest potential benefit.

**Anemia:** Another relevant observation concerns anemia, a common comorbidity in HF that contributes to disease progression via multiple mechanisms. MRAs, including finerenone, have been proposed to influence erythropoiesis through hepcidin inhibition. Despite this mechanistic rationale, patients treated with finerenone had slightly lower mean hemoglobin and hematocrit levels, with no meaningful clinical difference. Importantly, the treatment effect of finerenone on the primary outcome remained consistent regardless of anemia status (RR 0.89; 95% CI, 0.73–1.10 with anemia vs. RR 0.76; 95% CI, 0.64–0.91 without anemia; *p* for interaction = 0.27). However, no benefit was observed in terms of anemia resolution or prevention of new-onset anemia [62].

**Other Comorbidities:** Efficacy was also preserved in patients with chronic obstructive pulmonary disease (COPD) [63], dispelling concerns raised by the TOPCAT trial regarding an interaction with pulmonary disease status [64]. The cardiovascular protection was independent of diabetic status [65] or insulin resistance [66], supporting the expansion of finerenone’s indication beyond diabetic kidney disease.

### 5.4. Timing, Onset, and Concomitant Therapies

Mirroring the rapid kinetic profile observed with SGLT2 inhibitors, finerenone achieved statistical significance for the primary outcome as early as 28 days post-randomization (RR 0.62; 95% CI 0.40–0.97; *p* = 0.037), with a significant separation of event curves that persisted throughout the follow-up. This rapid onset suggests mechanisms beyond long-term anti-fibrotic remodeling, potentially involving hemodynamic load reduction or rapid anti-inflammatory effects [67]. This rapid action supports early initiation, particularly in vulnerable phases. Indeed, patients initiated on finerenone within 7 days of a worsening heart failure event showed a trend toward greater absolute risk reduction compared to stable outpatients (RR 0.74; 95% CI 0.57–0.95), although it did not achieve formal statistical significance (*p* for interaction = 0.07) [68]

In TOPCAT, conversely, spironolactone provided *less* benefit to patients enrolled within a year of an HF hospitalization.

Given that SGLT2 inhibitors are now a Class I recommendation for HFpEF, the interaction between finerenone and these agents is clinically paramount. Vaduganathan et al. demonstrated that finerenone provides an additive benefit on top of SGLT2 inhibitors without increasing adverse events (RR 0.83 with SGLT2i vs. 0.85 without; *p*-interaction = 0.76), supporting a potential additive therapeutic approach even in HFpEF [69].

Finally, the efficacy and safety profile of finerenone was observed to be consistent across patient subgroups categorized by baseline diuretic use (*p* for interaction = 0.18). Also, patients randomized to finerenone were significantly less likely to require an escalation in their diuretic dosage compared to the placebo cohort; conversely, they were more prone to a reduction or discontinuation. This finding is coherent with inhibition of sodium reabsorption in the distal nephron via a distinct mechanism that can augment the natriuresis induced by loop and thiazide diuretics, potentially reducing the required dosage of these agents. The reduced need for diuretic intensification is an important clinical finding, as it is widely recognized as a surrogate marker for worsening heart failure and is associated with poor clinical prognoses. This diuretic-sparing effect of finerenone showed early in the treatment period and was sustained throughout the follow-up, indicating that the benefits are likely attributable to a durable effect rather than an initial decongestive action only [70].

Prior to this, the therapeutic landscape for HFpEF and HFmrEF was dominated by a single drug class—SGLT2 inhibitors—which alone had demonstrated the capacity to lower cardiovascular mortality and heart failure events. The FINEARTS-HF trial is therefore the first study to establish that a nonsteroidal MRA, finerenone, can achieve this outcome, demonstrating consistent efficacy across a broad spectrum of clinical conditions and comorbidities. (Table 1).

## 6. Clinical Implications and Limitations

Finerenone’s current indication, according to the 2023 ESC heart failure guideline update, is a Class I recommendation for reducing HF risk in the specific population of patients with diabetes and CKD [2]. However, based on the robust evidence from FINEARTS-HF, finerenone is poised to redefine the standard of care, moving beyond its current restriction to diabetic kidney disease. It should now be considered a core pillar of GDMT for HFmrEF and HFpEF, ideally implemented alongside SGLT2 inhibitors. Patient selection can be strategic: while efficacy is broadly consistent, subgroups with obesity, albuminuria, or recent worsening heart failure events may derive the highest absolute risk reduction and should be prioritized. In clinical practice, the combination with SGLT2 inhibitors appears not only safe but mechanistically synergistic, addressing both hemodynamic load and myocardial fibrosis without amplifying adverse events. Safety monitoring remains a cornerstone of implementation; potassium levels should be checked properly, yet the risk of severe hyperkalemia is low and manageable. Clinicians should adopt a proactive approach, integrating finerenone early to maximize organ protection and significantly reduce the burden of rehospitalization.

Despite these promising findings, several limitations must be acknowledged. First, while finerenone significantly reduced worsening heart failure events, the reduction in cardiovascular death did not reach statistical significance (HR 0.93). However, this finding is not entirely unexpected in the HFpEF population, where mortality is frequently driven by non-cardiovascular comorbidities and competing causes rather than HF progression alone. Second, the absolute risk reduction for the primary outcome, though significant, was relatively modest (ARR 2.8 per 100 person-years). Finally, there remains a gap between the controlled environment of clinical trials and real-world practice; issues such as cost-effectiveness, polypharmacy burden, and actual adherence in routine care need to be carefully evaluated to ensure the trial’s benefits translate effectively into broader populations.

## 7. Future Directions and Unmet Needs

The field of MR modulation is rapidly evolving, yet several key questions remain. The large-scale Phase III BalanceD-HF trial (NCT06307652) will provide evidence on the efficacy of the MRM balcinrenone combined with dapagliflozin in a high-risk population of patients with HF and impaired kidney function. Besides Balcinrenone, the “MOONRAKER” clinical trial program is actively expanding the evidence base for finerenone in high-risk populations. The REDEFINE-HF trial (NCT06008197) is currently evaluating finerenone in patients hospitalized with acute decompensated heart failure (LVEF ≥ 40%), a setting where early initiation could prove crucial. Concurrently, the CONFIRMATION-HF trial (NCT06024746) is testing a combination strategy of finerenone plus an SGLT2 inhibitor versus usual care in hospitalized patients to determine the safety and efficacy of rapid, simultaneous guideline-directed medical therapy implementation. Finally, the FINALITY-HF trial (NCT06033950) addresses a significant unmet need by investigating finerenone in patients with HFrEF who are intolerant to or ineligible for steroidal MRAs, potentially offering a solution for those currently excluded from mineralocorticoid receptor antagonism.

Also, the SPIRIT-HF (NCT04727073) trial is re-evaluating the role of sMRAs in HFpEF, comparing spironolactone to placebo.

A significant evidence gap is the lack of direct, large-scale comparative trials between nsMRAs and sMRAs, making it difficult to definitively assess their relative efficacy and safety. Meta-analyses confirm the strong benefit of sMRAs in HFrEF and suggest a benefit for nsMRAs in HFmrEF/HFpEF [72], but direct comparisons are confounded by differences in trial populations and evolving standards of care over time in the older sMRA trials and more contemporary nsMRA trials.

Finally, practical challenges such as the historically low clinical uptake of sMRAs raise questions about whether the improved tolerability of non-steroidal agents will translate to broader clinical adoption. Further research is needed to determine if nsMRAs or other emerging therapies offer superior efficacy, safety, or tolerability also in HFrEF and to establish their efficacy in broader HF populations.

## 8. Conclusions

MRAs represent a therapeutic approach that maintains its relevance not only in patients with HFrEF but also in those with HFmrEF and, potentially, in patients with HFpEF. While, indeed, the results of the TOPCAT trial showed a reduction in cardiovascular events only at the limits of statistical significance, subgroup analysis suggested that certain clinical phenotypes of patients with HFpEF might benefit more from the use of MRAs. Specifically, in patients with obesity, hypertension with concentric hypertrophy resistant to therapy, or with systemic inflammation, MRAs appear to exert greater efficacy. Finerenone’s distinct pharmacodynamic and pharmacokinetic properties set it apart from other MRAs, endowing it with a favorable safety and clinical efficacy profile. This was first established by its reduction of cardiorenal outcomes in the setting of diabetic kidney disease. Building on this, the Phase 3 FINEARTS-HF trial was positioned to address the limitations of TOPCAT. Its positive results—demonstrating efficacy versus placebo in reducing cardiovascular mortality and heart failure events among patients with an LVEF of 40% or higher—have now changed the management landscape, offering an additional therapeutic opportunity for this population. While the 2023 ESC guidelines currently limit the Class I recommendation for finerenone to the reduction of heart failure risk in patients with type 2 diabetes and CKD, the FINEARTS-HF evidence strongly advocates for an expanded indication. This supports the integration of finerenone as a core therapeutic pillar, complementary to SGLT2 inhibitors, for the management of HFmrEF and HFpEF.

The journey from the non-selective steroidal MRAs to the highly targeted non-steroidal MRAs and the conceptually advanced mineralocorticoid receptor modulators and aldosterone synthase inhibitors reflects a remarkable evolution in our understanding of the mineralocorticoid receptor axis and our ability to pharmacologically modulate it. The future of cardiorenal therapy will likely be characterized by a personalized, combined approach to tailor treatment to an individual patient’s specific risk profile, thereby maximizing organ protection while minimizing adverse effects.

## Figures and Tables

**Table 1:** Prespecified and Subgroup Analyses of FINEARTS-HF Trial.

Author	Focus of Analysis/Target Population/Subgroup	Key Findings & Outcomes	Clinical Implications
Vaduganathan M. et al., 2024 [46]	Projected lifetime effects in total study cohort (N = 6001)	Estimated gain of 3.1 event-free years in 55-year-olds (95% CI 0.8–5.4; *p* = 0.007) and 2.0 years in 65-year-olds (95% CI 0.8–3.3; *p* < 0.001)	Early initiation of therapy correlates with maximized cumulative survival benefit, particularly in younger to middle-aged patients
Docherty K.F. et al., 2024 [47]	Efficacy stratified by LVEF: <50% vs. 50–60% vs. ≥60%; Patients with available baseline LVEF (N = 5993)	Consistent risk reduction across all strata: RR 0.84 (LVEF < 50%), 0.80 (LVEF 50–60%), 0.94 (LVEF ≥ 60%). No significant interaction (*p* = 0.70)	Efficacy is preserved regardless of ejection fraction, supporting broad applicability across the HFmrEF/HFpEF continuum
Vaduganathan M. et al., 2024 [69]	Efficacy and safety based on concomitant SGLT2i use; Users (N = 817) vs. Non-users (N = 817) of SGLT2i at baseline	Similar magnitude of benefit in patients on SGLT2i (HR 0.83) versus those not on therapy (HR 0.85); *p*-interaction = 0.76.	Finerenone provides additive benefit on top of standard-of-care SGLT2i therapy without safety signals
Chimura M. et al., 2024 [54]	Efficacy by age stratification; age quartiles (Q1 to Q4)	Uniform efficacy across age groups (*p*-interaction = 0.27). HR ranged from 0.70 in youngest to 0.85 in oldest quartile.	Advanced age does not diminish therapeutic efficacy or increase the rate of adverse events significantly.
Vaduganathan M. et al., 2024 [67]	Onset of benefit as time-to-event analysis; total study cohort (N = 6001)	Significant divergence in primary endpoint curves observed by day 28 (HR 0.62; *p* = 0.037).	Rapid therapeutic onset suggests mechanisms involving hemodynamic load reduction beyond slow anti-fibrotic remodeling.
Mc Causland F.R. et al., 2024 [45]	Renal Outcome Analysis; total study cohort (N = 6001)	No significant difference in composite renal endpoints (HR 1.33). Marked reduction in new-onset microalbuminuria (−24%) and macroalbuminuria (−38%).	While not affecting eGFR decline slopes in this low-risk population, the drug offers significant anti-albuminuric protection.
Desai A.S. et al., 2024 [68]	Efficacy and safety stratified by time from WHF event; total study cohort (N = 6001)	Trend toward greater relative risk reduction in patients enrolled <7 days post-discharge (HR 0.74) vs. >3 months (HR 0.99).	Initiating therapy in the vulnerable post-discharge phase may yield superior absolute risk reductions.
Yang M. et al., 2024 [56]	Efficacy stratified by baseline KCCQ-TSS; baseline KCCQ-TSS available in 5986 patients	Consistent event reduction across symptom tertiles (*p*-interaction = 0.89). Significant improvement in KCCQ scores at 1 year (+1.6 points; *p* < 0.001).	Improving quality of life is a distinct benefit, achievable regardless of baseline symptom severity.
Chimura M. et al., 2024 [53]	Efficacy and safety by sex; total study cohort (N = 6001); Male vs. Female	Comparable efficacy in women (HR 0.78) and men (HR 0.88); *p*-interaction = 0.41. Similar safety profile.	Sex does not modulate the therapeutic response, confirming utility in the female-predominant HFpEF population.
Matsumoto S. et al., 2025 [71]	Efficacy and safety based on baseline AF status; Paroxysmal 1384 (23.1%) vs. Persistent 1886 (31.5%) vs. No AF	Efficacy maintained across AF subtypes (*p*-interaction = 0.94). Trend towards reduction in new-onset AF (HR 0.77; *p* = 0.09).	Investigational signal suggesting potential anti-arrhythmic properties mediated by atrial reverse remodeling
Butt JV et al., 2025 [65]	Efficacy safety analysis by Glycemic Status; Diabetes 2764 (46.2%) vs. Pre-diabetes 1979 (33.1%) vs. Normoglycemia 1243 (20.8%)	Consistent benefit across glycemic spectrum (*p*-interaction = 0.93). HR ranged from 0.82 (diabetes) to 0.85 (normoglycemia)	Cardiovascular protection is independent of diabetic status, expanding indication beyond diabetic kidney disease.
Ostrominski, JW et al., 2025 [57]	Efficacy/safety by NYHA class; NYHA II 4146 (69%) vs. III/IV 1854 (31%)	Relative benefit consistent (*p* = 0.54), but absolute risk reduction more than double in NYHA III/IV (ARR 4.5 vs. 2.0 per 100 py).	Treating more symptomatic patients yields a higher return in terms of absolute events prevented.
Cunningham JW et al., 2025 [48]	Influence of baseline NT-proBNP; NT-proBNP available in 5843 patients	Baseline natriuretic peptide levels did not predict or alter the treatment response (*p* interaction = 0.92). Finerenone significantly lowered NT-proBNP levels by ~12% at both 3 and 12 months	Influence of baseline NT-proBNP; NT-proBNP available in 5843 patients
Chimura M et al., 2025 [62]	Effects according to anaemia status; 1584 (28.0%) with baseline anaemia	Clinical efficacy was not hindered by the presence of anaemia (RR 0.89 in anaemic vs. 0.76 in non-anaemic patients; *p* interaction = 0.27)	Mean haemoglobin levels showed a slight, non-clinical decrease (−0.12 g/dL) in the treatment arm compared to placebo
Pabon MA et al., 2025 [51]	Efficacy/safety in participants with HFimpEF; 273 (5%) patients with a history of LVEF < 40%	Efficacy in HFimpEF (HR consistent with main cohort, *p*-interaction = 0.36). Higher absolute risk reduction (9.2 events per 100 py).	Patients with recovered EF remain at high risk and derive substantial benefit from continued neurohormonal blockade
Butt JH et al., 2025 [63]	Effects according to COPD status/773 (12.9%) with COPD	risk reduction was identical in patients with and without COPD (RR 0.84; *p* interaction = 0.93)	Comorbid COPD did not modify the efficacy or safety outcomes of finerenone
Butt JH et al., 2025 [55]	Effects according to frailty status/Frailty index: not frail (27%), more frail (36%), most frail (37%)	Benefits regarding the primary outcome remained consistent across the entire spectrum of frailty (*p* interaction = 0.77)	Frailty status was not a modifier for either the safety or efficacy of the drug
Chimura M et al., 2025 [70]	Efficacy/tolerability by background diuretic therapy/Non-loop (12.6%), Loop ≤ 40 mg (56%), Loop > 40 mg (21%), Combined (10%)	No interaction was found between baseline diuretic intensity and treatment effect (*p* interaction = 0.18). Furthermore, finerenone therapy reduced the likelihood of needing loop diuretic dose escalation	The type and dose of background diuretics did not influence finerenone’s performance or safety profile
Ostrominski JW et al., 2025 [60]	Analysis by baseline KDIGO kidney risk/Low (35%), Moderate (29%), High/Very High (36%) risk	Clinical efficacy on outcomes and symptoms was maintained across all kidney risk categories (*p* interaction = 0.24). Reduction in UACR was more pronounced in higher-risk groups	Baseline renal risk profile did not modify clinical results, though antiproteinuric effects were greater in patients with higher baseline risk
Ostrominski JW et al., 2025 [66]	Analysis based on insulin resistance (eGDR)/5851 (98%) with calculable eGDR	The benefits on CV death and total HF events were consistent across estimated glucose disposal rate (eGDR) categories (*p* interaction = 0.64), as was the effect on new-onset diabetes (*p* = 0.36)	Insulin resistance status did not act as a modifier for the drug’s safety or efficacy

AF, Atrial Fibrillation; ARR, absolute rate reduction; CI, Confidence Interval; COPD, Chronic Obstructive Pulmonary Disease; eGDR, estimated glucose disposal rate; eGFR, estimated Glomerular Filtration Rate; HFimpEF, Heart Failure with Improved Ejection Fraction; HFmrEF, Heart Failure with mildly reduced Ejection Fraction; HFpEF, Heart Failure with preserved Ejection Fraction; HR, hazard ratio; KCCQ-TSS, Kansas City Cardiomyopathy Questionnaire Total Symptom Score; KDIGO, Kidney Disease Improving Global Outcomes; LVEF, Left Ventricular Ejection Fraction; UACR, urine albumin-to-creatinine ratio; WHF, Worsening Heart Failure.
